# Factors associated with dietary supplement use in Saudi pregnant women

**DOI:** 10.1186/s12978-017-0357-7

**Published:** 2017-08-29

**Authors:** Hanan A Alfawaz, Nasiruddin Khan, Najlaa AlOteabi, Syed D. Hussain, Nasser M. Al-Daghri

**Affiliations:** 1 Department of Food Science and Nutrition, College of Food Science and Agriculture, King Saud University, Riyadh, 11451 Saudi Arabia; 2 Department of Food Science and Human Nutrition, Human Nutrition College of Applied Science, A’Sharqiyah University, 400 Ibra, Oman; 3 Biochemistry Department, College of Science, Prince Mutaib Chair for Biomarkers of Osteoporosis, King Saud University, Riyadh, 11451 Saudi Arabia

**Keywords:** Dietary supplement, Attitude, Behavior, Socio-demographic, Saudi Arabia

## Abstract

**Background:**

The aim was to investigate the prevalence of dietary supplement use among pregnant Saudi women and its associations between various demographics.

**Methods:**

In this cross-sectional study, a total of 137 pregnant women attending prenatal care from King Salman Hospital completed a self-administered questionnaire including socio-demographic characteristics, general awareness, attitude and behavior towards use of dietary supplements during pregnancy.

**Results:**

Dietary supplement use among Saudi women in pregnancy was high (71.5%) and was significantly associated with level of education (*p* = 0.005), family income (*p* = 0.039) and number of children (*p* = 0.007). No significant association was observed between neonatal health outcomes and dietary supplement use during pregnancy. In all participants, 81.6% believed that supplement use is important for nutritional status and more favorable neonatal outcomes. For the majority of participants, the primary source of information for dietary supplement use was a doctor’s advice. The majority of the participants [65.7% (*n* = 90)] responded that dietary supplement use is safe. Folic acid was found to be the most common type of dietary supplement used (95.9%; *n* = 94); however, 53.1% (*n* = 52) did not take folic acid supplements 3 months prior to pregnancy. Other common supplements used were iron, calcium and vitamin D (88.8, 81.6, and 41%, respectively).

**Conclusions:**

This study provided new information on dietary supplement use and its correlates in Saudi pregnant women. The prevalence of dietary supplement use was high in this group and was associated with socio-demographic and lifestyle characteristics.

## Plain English summary

There is limited data available on the use of dietary supplement among pregnant Arab women. The present study aims to fill this gap. A total of 137 pregnant women from King Salman Hospital were recruited and completed a questionnaire. Results indicated that use of dietary supplements during pregnancy was high (71.5%) and significantly associated with level of education, family income and number of children. No significant association was observed between infant health outcomes and dietary supplement use during pregnancy. The majority (>80%) of participants acknowledge the importance of supplement use to compensate for increased metabolic demands of pregnancy. Doctor’s advice was the main source of information and 65.7% (*n* = 90) believed that dietary supplement are safe. Folic acid was the most common type of dietary supplement use (95.9%; *n* = 94), followed by iron, calcium and vitamin D (88.8, 81.6, and 41%, respectively). This study provided new information on the dietary supplement use and its correlates in Saudi pregnant women. The prevalence of dietary supplement use was high in this group and was significantly associated with differences in socio-demographic and lifestyle characteristics.

## Background

The fast economic growth of Saudi Arabia in the last few decades has greatly affected the cultural diet and lifestyle of its general population. Energy-dense Western diets have replaced the traditional Saudi diet, which, in combination with a sedentary lifestyle, has led to an increased prevalence of non-communicable diseases such as obesity, type 2 diabetes mellitus and hypertension [1–7]. Furthermore, according to the World Health Organization (WHO), the most affected population from poor dietary habits and malnutrition are children, adolescents and women of reproductive age [8].

With the rising burden of diseases, Saudi Arabia is one of the largest pharmaceutical markets in the Arab region [9–11]. Furthermore, the well-established vitamin and supplement market in the country accounts for 4% of the total pharmaceutical market sales (US $80 M) [12]. Many recent studies observed the lack of knowledge, especially in girls regarding micronutrients [13, 14] and supplement intake among pregnant Saudi women from various regions [15–17]. Moreover, a study performed by Gemeda Daba et al. has demonstrated a significant positive relationship between nutritional information and level of education among pregnant women [18]. However, there is scarcity of data regarding the prevalence of supplement use in Saudi pregnant women and the relationship with various socio-demographic factors, attitudes, behavior and awareness.

## Methods

### Study population

In this cross-sectional study, 200 Saudi pregnant women in their second or third trimester were recruited, out of whom 137 consented and completed the questionnaire. The participants were recruited from the obstetric clinics in King Salman Hospital, Riyadh, Saudi Arabia.

### Data collection and measurements

A pilot study of 10 pregnant women was performed to confirm the reliability and validity of the questionnaire. Content and face validity were done by health professionals and physicians regarding the clarity in all questions. The questionnaire was then reviewed by experts in the related fields. Moreover, external reviewers provided their feedback and opinion in developing/improving the questionnaire to ensure reliability of the test. From the pilot study, the prevalence of supplement use was 75.0% among pregnant women. To achieve 95% confidence intervals and 8% absolute error margin, 113 pregnant women were required. To overcome non-response 200 pregnant women were recruited.

Expert feedback and suggestions were incorporated in the final questionnaire. Furthermore, Cronbach’s α, an estimate of coefficient of reliability, 84% was measured for the questionnaire.

The participants were asked to complete the self-administered questionnaire which was divided into four parts: (1) socio-demographic and lifestyle characteristics (less than 5000 Saudi Arabia riyals (SAR) was considered low income, between 5000 and 9999 SAR was considered average income 10,000–16,000 SAR was considered moderate income and more than 16,000 SAR was considered high income), (2) history of disease and prevalence of dietary supplement (DS) use, (3) reasons, duration, frequency and source of knowledge of dietary supplements, and (4) awareness and attitudes about supplement use and common types of dietary supplements used before and during pregnancy. The questionnaire also included the source of spending, and the circumstances surrounding use of dietary supplements.

### Data analysis

Data was analyzed using the Statistical Package for Social Sciences (SPSS) 22.0 (SPSS Inc., Chicago, IL, USA). Data was presented as frequencies (%). Pearson Chi-square test was used to examine differences between use of dietary supplements during pregnancy and body mass index (BMI), educational level, family income, occupation, number of children and neonatal health. All *p*-values were two-tailed, and *p*-values <0.05 were significant.

## Results

Table 1 shows the socio-demographic/lifestyle characteristics of the participants (*n* = 137). The majority of the participants had a normal BMI (<25 kg/m^2^) (*N* = 52; 38.2%), with average family income (*N* = 55; 40.1%), and most held college degrees (*N* = 93; 67.9%). The percentage of women employed and housewives were (*N* = 58; 42.3%), and (*N* = 56; 40.9%), respectively.

**Table 1:** Use of Dietary Supplements during Pregnancy based on Sociodemographic/Lifestyle Characteristics

Parameters	N (%)	Dietary Supplement Use during Pregnancy		*P*-values
		Yes	No	
BMI (kg/m^2^) Status				0.72
Underweight (<19.5)	7 (5.1)	6 (6.2)	1 (7.7)	
Normal (19.5–24.9)	52 (38.2)	37 (38.1)	6 (46.2)	
Overweight (25–29.9)	46 (33.8)	36 (37.1)	3 (23.1)	
Obese (≥30)	31 (22.8)	18 (18.6)	3 (23.1)	
Educational Level				0.005
Intermediate or less	6 (4.4)	4 (4.1)	2 (15.4)	
High School	16 (11.7)	9 (9.2)	5 (38.5)	
Bachelor	93 (67.9)	70 (71.4)	6 (46.2)	
Post Graduate	22 (16.1)	15 (15.3)	0 (0.0)	
Family Income (SAR)				0.039
less than 5000	19 (13.9)	13 (13.3)	5 (38.5)	
5000–9999	55 (40.1)	44 (44.9)	3 (23.1)	
10,000–16,000	35 (25.5)	24 (24.5)	1 (7.7)	
> 16,000	28 (20.4)	17 (17.3)	4 (30.8)	
Occupation				0.79
Employee	58 (42.3)	46 (46.9)	5 (38.5)	
Housewife	56 (40.9)	38 (38.8)	6 (46.2)	
Student	23 (16.8)	14 (14.3)	2 (15.4)	
Number of children				0.007
2 and less	78 (56.9)	61 (62.2)	3 (23.1)	
3 to 5	46 (33.6)	30 (30.6)	7 (53.8)	
6 to 8	12 (8.8)	7 (7.1)	2 (15.4)	
> 8	1 (0.7)	0 (0.0)	1 (7.7)	

Data presented as frequencies (valid %)

The participant’s history of disease and prevalence of dietary supplements use before and during pregnancy are presented in Table 2. Among all the participants, about 62.2% self-reported that they were vitamin D deficient.

**Table 2:** History of Disease and Prevalence of Dietary Supplements

Participants History	N (%)
Do you any of the following health problems?	
Anemia	34 (29.3)
Diabetes Mellitus	6 (4.4)
Dyslipidemia (High Cholesterol / High Triglycerides)	13 (9.7)
Hypertension	8 (6.1)
Osteoporosis	7 (5.3)
Thyroid Disorder	14 (10.7)
Vitamin D Deficiency	74 (62.2)
Have you ever checked your vitamin status?	
Yes	76 (57.6)
No	56 (42.4)
Do you take supplements when you are pregnant?	
Yes	98 (71.5)
No	13 (9.5)
Sometimes	26 (19.0)
If yes, do you read the supplement’s label before using?	
Yes	67 (68.4)
No	28 (28.6)
Sometimes	3 (3.1)
Do you take supplements throughout pregnancy?	
Yes	66 (67.3)
No	23 (23.5)
Sometimes	9 (9.2)

Data presented as frequencies (%)

The association between use of dietary supplements, BMI, educational level, family income, occupation and number of children are presented in Table 1. The frequency of using dietary supplements during pregnancy was high and significantly associated with level of education (*p* = 0.005), family income (*p* = 0.039) and number of children (*p* = 0.007) as compared to non-users. There were no significant differences in the incidence of rickets, walking delays/appearance of teeth, weak or late growth, spina bifida, diabetes mellitus and allergies between those who are taking dietary supplement and those who don’t (not shown in table). The duration of supplement use, reasons, frequency, and source of knowledge is shown in Table 3. Among all participants, 81.6% believed that their diet was insufficient for good health in pregnancy and use of supplements is important. The main reasons for the use of supplements in pregnancy was due to poor maternal nutritional status and neonatal requirements. The majority of participants described the source of information regarding dietary supplements was a doctor.

**Table 3:** Perceptions and behavior related of dietary supplement use among Saudi women

Survey Question	N (%)
Do you think your diet is sufficient and you don’t need supplements?	
Yes	7 (7.1)
No	80 (81.6)
Sometimes	11 (11.2)
What are your reasons for using dietary supplements?^a^	
Poor nutritional status	71 (72.4)
Baby requirements exceed my usual diet	72 (73.5)
I have nutritional deficiency	68 (69.4)
How long have you been using supplements?	
Less than 3 months	30 (30.6)
3 Months	23 (23.5)
6 Months	28 (28.6)
9 Months	32 (32.7)
12 Months	7 (7.1)
More than a year	14 (14.3)
Sources of information about vitamins and supplements^a^	
Attending physician	90 (91.8)
Friend’s advice	8 (8.2)
Internet	14 (14.3)
Social Media	7 (7.1)
Sources of Spending^a^	
My expense	54 (55.1)
Free	31 (31.6)
Insurance	24 (24.5)

Data presented as frequencies (%); ^a^indicates questions with multiple responses

Table 4 shows the awareness and attitudes about dietary supplement use, most participants 65.7% (*N* = 90) responded that they are safe to use. About 53.1% (*N* = 52) did not take folic acid supplements 3 months prior to pregnancy. Folic acid was found to be the most common dietary supplements used among pregnant women (95.9%; *N* = 94) followed by iron, calcium and vitamin D (88.8, 81.6 and 41%, respectively).

**Table 4:** Awareness and Attitudes about Dietary Supplements used before and during Pregnancy

Survey Questions	Yes	No	Sometimes
Do you think using dietary supplement is safe?	90 (65.7)	18 (13.1)	29 (21.2)
Do you take folic acid before pregnancy?	44 (44.9)	52 (53.1)	2 (2.0)
Do you take the following dietary supplements during pregnancy?			
Calcium	80 (81.6)	16 (16.3)	2 (2.0)
Folic acid	94 (95.9)	4 (4.1)	0 (0)
Iodine	27 (27.6)	69 (70.4)	2 (2.0)
Iron	87 (88.8)	10 (10.2)	1 (1.0)
Vitamin B Complex	40 (40.8)	51 (52.0)	7 (7.1)
Vitamin D	41 (41.8)	53 (54.1)	4 (4.1)

Data presented as frequencies (%)

## Discussion

The present study is the first of its kind to demonstrate the high prevalence (71.5%) of dietary supplement use among Saudi women in pregnancy. There was no significant association between health problems in babies and dietary supplement use in pregnancy. Number of children, monthly income and education level were significantly associated with supplement use as compared to non-users. There is a need to improve the level of awareness and attitudes about supplement use of common dietary supplements in pregnancy.

The prevalence of dietary supplement use in the general population has been reported in different parts of Saudi Arabia [19]. In addition, use of dietary supplements in pregnant Saudi women had been reported in several recent studies [15, 16, 20, 21]. The high prevalence (71.5%) of dietary supplement use in the present study reinforces these local findings. Furthermore, Aronsson and colleagues demonstrated that 92% of the 7326 women (from USA, Sweden, Finland, and Germany) were using one or more types of supplement during pregnancy [20]. Another study by Pouchieu and colleagues [21] showed a high proportion of pregnant women (64.9%) in France used dietary supplements at least 3 days a week. The frequency of using dietary supplements during pregnancy was high and significantly associated with level of education, family income and number of children as compared to non-users. There are various studies from developed countries showing a positive association of dietary supplement use and socio-economic status during pregnancy [22–25]. The above mentioned studies [20, 21] also showed a direct correlation of dietary supplement use in pregnant women with higher income and educational status. The present study is consistent with these studies [20, 21] showing a direct association between dietary supplement use with level of education and family income. Other studies have shown an inverse association between the number of children and use of dietary supplements in pregnancy [25–27]. However, Foote and colleague showed opposite results in that the number of children was not associated with dietary supplement use, in healthy women [28]. Pouchieu and colleagues demonstrated that women with children used less dietary supplements due to fewer physician consultations [21]. This finding was in accordance with the present result showing that women who had more children were used less dietary supplements as compared to those who have less children.

With regards to other micronutrients, vitamin D and iodine deficiencies have been associated with various fetal and maternal abnormalities [29, 30]. The prevalence of vitamin D deficiency and insufficiency (50.0 and 43.8%, respectively) has been reported among pregnant Saudi women with adequate vitamin D intake (≥600 IU/day) among only 8.1% of pregnant women [14, 31, 32].

The most common type of dietary supplement used however in pregnant women was found to be folic acid followed by iron and calcium. Among participants, 44.9% (*n* = 44) women took folic acid supplement 3 months before pregnancy, while 95.9% (*n* = 94) took folic acid only during pregnancy. These results are not in accordance with other studies performed in Saudi Arabia [15, 16] and maybe due to the different levels of awareness among pregnant patients recruited to include educational level and the number of times the patient got pregnant. Despite discrepancy, the figures correspond to reports from the Netherlands [33] which showed the pre-conception intake of folic acid was up to 39%.

It is known that the need for specific micronutrients such as folic acid increases during pregnancy [34]. For instance, 0.4 mg/day of folic acid is recommended before conception to minimize the risk of neural tube defects and megaloblastic anemia [35]. Recent studies showed that the prevalence and use of folic acid in Saudi women was between 6.8–9.7% based on its use during and before pregnancy [16]. A study performed by McWalter et al. showed that the prevalence of folic acid supplementation in pregnant Saudi women was 10% before 3 months and 3% (*n* = 168) at 6 months of gestation [15].

The authors acknowledge some limitations of this study. First, the findings cannot be generalized due to small but adequate sample size, but because qualitative findings by nature are not generalizable, we do aim for transferability. Second, the list of supplements included was not comprehensive, thus possibly missing out other adjunct dietary influences. Lastly, age was not included in the questionnaire and hence further analysis on the influence of age on dietary supplement use is not included in the present study.

## Conclusion

This study demonstrated new information on the use of different dietary supplements use during pregnancy as well as their relationship with various demographic factors and general awareness and attitude. Based on these results, the main reason behind using dietary supplements during or before pregnancy was to compensate for poor nutritional imbalance and neonatal requirements owing to increased metabolic demands of pregnancy. It appears necessary to increase awareness among pregnant women regarding the appropriate use of different dietary supplements before, after and during pregnancy. It is recommended to educate the general population through media and health professionals and work towards implementation of national recommendations of dietary supplement use during pregnancy.

## Abbreviations

BMI: Body mass index
DS: Dietary supplements
SAR: Saudi Arabian Riyal
SPSS: Statistical Package for Social Sciences
WHO: World Health Organization
